# Cropping systems modulate the rate and magnitude of soil microbial autotrophic CO_2_ fixation in soil

**DOI:** 10.3389/fmicb.2015.00379

**Published:** 2015-05-08

**Authors:** Xiaohong Wu, Tida Ge, Wei Wang, Hongzhao Yuan, Carl-Eric Wegner, Zhenke Zhu, Andrew S. Whiteley, Jinshui Wu

**Affiliations:** ^1^Key Laboratory of Agro-ecological Processes in Subtropical Region and Changsha Research Station for Agricultural and Environmental Monitoring, Institute of Subtropical Agriculture, Chinese Academy of SciencesChangsha, China; ^2^ISA-CAS and UWA Joint Laboratory for Soil Systems BiologyChangsha, China; ^3^Department of Biogeochemistry, Max Planck Institute for Terrestrial MicrobiologyMarburg, Germany; ^4^School of Earth and Environment, The University of Western AustraliaCrawley, WA, Australia

**Keywords:** cropping systems, autotrophic bacteria CO_2_ fixation, RubisCO, *cbbL* genes, ^14^C continuous labeling, ^14^C-SOC, soil depth

## Abstract

The effect of different cropping systems on CO_2_ fixation by soil microorganisms was studied by comparing soils from three exemplary cropping systems after 10 years of agricultural practice. Studied cropping systems included: continuous cropping of paddy rice (rice-rice), rotation of paddy rice and rapeseed (rice-rapeseed), and rotated cropping of rapeseed and corn (rapeseed-corn). Soils from different cropping systems were incubated with continuous ^14^C-CO_2_ labeling for 110 days. The CO_2_-fixing bacterial communities were investigated by analyzing the *cbbL* gene encoding ribulose-1,5-bisphosphate carboxylase oxygenase (RubisCO). Abundance, diversity and activity of *cbbL*-carrying bacteria were analyzed by quantitative PCR, *cbbL* clone libraries and enzyme assays. After 110 days incubation, substantial amounts of ^14^C-CO_2_ were incorporated into soil organic carbon (^14^C-SOC) and microbial biomass carbon (^14^C-MBC). Rice-rice rotated soil showed stronger incorporation rates when looking at ^14^C-SOC and ^14^C-MBC contents. These differences in incorporation rates were also reflected by determined RubisCO activities. ^14^C-MBC, *cbbL* gene abundances and RubisCO activity were found to correlate significantly with ^14^C-SOC, indicating *cbbL-carrying* bacteria to be key players for CO_2_ fixation in these soils. The analysis of clone libraries revealed distinct *cbbL*-carrying bacterial communities for the individual soils analyzed. Most of the identified operational taxonomic units (OTU) were related to *Nitrobacter hamburgensis, Methylibium petroleiphilum, Rhodoblastus acidophilus, Bradyrhizobium, Cupriavidus metallidurans, Rubrivivax, Burkholderia, Stappia, and Thiobacillus thiophilus*. OTUs related to *Rubrivivax gelatinosus* were specific for rice-rice soil. OTUs linked to *Methylibium petroleiphilum* were exclusively found in rice-rapeseed soil. Observed differences could be linked to differences in soil parameters such as SOC. We conclude that the long-term application of cropping systems alters underlying soil parameters, which in turn selects for distinct autotrophic communities.

## Introduction

Autotrophic bacteria with the capacity to fix CO_2_ are widespread in extreme terrestrial ecosystems (Giri et al., 2004; Nanba et al., 2004; Nakai et al., 2012). Recently, an isotope incubation experiment revealed high CO_2_ assimilation rates by autotrophic bacteria in agricultural soils, which represented a potential carbon sequestration of 0.6–4.9 Pg C year^−1^ (Yuan et al., 2012a; Ge et al., 2013). Autotrophic bacteria evolved six pathways for CO_2_ fixation: (1) the Calvin-Benson-Bassham cycle, (2) the reductive tricarboxylic acid cycle, (3) the reductive acetyl-CoA pathway, (4) the 3-hydroxypropionate cycle, (5) the 3-hydroxypropionate/4-hydroxybutyrate pathway, and (6) the dicarboxylate/4-hydroxybutyrate cycle (Fuchs, 2011). The predominant pathway for autotrophic bacteria to assimilate CO_2_ is the Calvin-Benson-Bassham cycle (CBB) (Selesi et al., 2005). Ribulose-1,5-bisphosphate carboxylase oxygenase (RuBisCO), the enzyme which catalyzes the rate-limiting step in the CBB cycle, exists in four distinct holoenzyme forms (I, II, III, and IV). These forms differ in structure, catalytic activity, and O_2_ sensitivity (Tabita, 1999). Form I RubisCO, composed of eight large subunits and eight small subunits, is the most abundant among the four forms (Tabita et al., 2008). Four clades of the *cbbL* gene that encodes the large subunits of form I RubisCO are known, namely IA to ID (Tabita, 1999). The presence of the *cbbL* gene has been documented in diverse phylogenetic groups from obligate autotrophic bacteria (form IA) to facultative autotrophic bacteria (form IC) (Kusian and Bowien, 1997; Kong et al., 2012).

In recent years, the *cbbL* gene has been widely used as a functional marker to analyze the diversity of autotrophic bacteria in diverse environments. Based on *cbbL* gene analysis, an unexpected level of *cbbL* diversity has been reported in agricultural soils (Selesi et al., 2005; Tolli and King, 2005; Yuan et al., 2012b; Xiao et al., 2014a). Phylogenetic analysis showed that *Azospirillum lipoferum, Rhodopseudomonas palustris, Bradyrhizobium japonicum, Ralstonia eutropha* are the dominant autotrophic bacteria in these soils (Yuan et al., 2012a). Different management practices including fertilizer treatments, land use alterations and different plant covers showed effects on the diversity and abundance of autotrophic bacterial communities in soils (Selesi et al., 2005; Tolli and King, 2005; Yuan et al., 2012b; Xiao et al., 2014a). Moreover, a link between microbial autotrophy and edaphic factors such as soil organic carbon (SOC), pH and clay content was identified (Selesi et al., 2007; Yuan et al., 2012a,b; Xiao et al., 2014a,b). A recent study revealed that differences in community composition, abundance and activity of autotrophic bacteria affect microbial carbon fixation across soil depth (Wu et al., 2014). A large proportion of the fixed C was restricted to surface soil (0–1 cm), and the assimilated ^14^C was mainly aliphatically stabilized in the humin fraction of agricultural soils (Hart et al., 2013a,b; Jian et al., 2014). Relating to agricultural soils, previous studies focused on the process of CO_2_ fixation by autotrophic bacteria in continuous cropping systems. However, information on autotrophic bacteria involved in CO_2_ fixation in rotated cropping systems is limited.

The rotation of paddy rice and upland crop is a common agricultural practice in the subtropical area of China (Zhu et al., 2010). Field studies indicated that paddy-upland rotated soils are characterized by different physical and chemical properties in comparison to paddy and upland soils (Nishida et al., 2013; Liu et al., 2014). The different soil condition in paddy-upland rotated soil was shown to affect soil microbial communities, especially functional guilds like purple phototrophic bacteria and methanogens (Feng et al., 2011; Bernard et al., 2012). The effect of paddy-upland rotation on autotrophic bacteria remains unclear. Therefore, three cropping systems: (i) double cropping of paddy rice, (ii) rotation of paddy rice and rapeseed, and (iii) double cropping of rapeseed and corn in an experimental field with a known cultivation record of continuous paddy rice farming were examined to study how different cropping systems affect CO_2_ fixation by autotrophic bacteria. Continuous ^14^C-CO_2_ labeling was applied to quantify the incorporation of microbial fixed C to the soil organic matter pool (^14^C-SOC) and soil microbial biomass (^14^C-MBC) at different depths (0–1, 1–5, 5–17 cm). Based on *cbbL* gene analysis, the abundance, diversity and composition of autotrophic bacterial communities in different cropping systems were investigated.

## Materials and methods

### Soils and sampling

The sampling was conducted in a long term agricultural management experiment site at Pantang in subtropical China (29° 10′−29° 18′ N, 111° 18′−111° 33′ E). The experimental site was characterized by a typical subtropical climate with an annual mean precipitation of approximately 1400 mm and an average annual temperature of 16.8°C. Three cropping systems, namely double cropping of paddy rice (rice-rice), rotation of paddy rice and rapeseed (rice-rapeseed), and double cropping of rapeseed and corn (rapeseed-corn) were established in 2000. Four replicate plots of each cropping system were randomly arranged in the fields. Soils at the field site were developed from quaternary red earth and were used for rice farming for decades prior to the implementation of the cropping systems. For rice-rice, the field was permanently flooded during the spring and autumn rice growing seasons. For rice-rapeseed, the field was flooded in the spring rice growing season while it was drained in the rapeseed season. For rapeseed-corn, crops were planted in rapeseed-corn sequence in a drained paddy field. These treatments were maintained for more than 10 years when we conducted this study. After the harvest of the late crop, one soil core was retrieved from each field by inserting a PVC column (10 cm diameter, 20 cm height) to 17 cm depth at a random location within each 33 m^2^ plot. Visible crops or grass at the surface were removed. Basic geochemical parameters for all soils are given in Table 1.

**Table 1:** **Characteristics of soils from different cropping systems**.

**Cropping system**	**pH**	**SOC (g kg^−1^)**	**Total N (g kg^−1^)**	**Total P (g kg^−1^)**	**Clay content (%)**	**CEC (cmol kg^−1^)**
Rice-rice	5.66 ± 0.01	20.93 ± 0.72	2.81 ± 0.01	0.70 ± 0.00	33.19 ± 0.43	13.16 ± 0.23
Rice-rapeseed	5.79 ± 0.01	6.64 ± 0.24	1.44 ± 0.00	0.82 ± 0.03	46.19 ± 0.41	7.96 ± 0.13
Rapeseed-corn	4.40 ± 0.03	6.19 ± 0.04	1.39 ± 0.01	0.75 ± 0.02	31.38 ± 0.55	11.05 ± 0.01

*Cropping systems were established in 2000*.

### Incubation with ^14^C-CO_2_

All PVC columns were incubated in a growth chamber (80 × 250 cm, height 120 cm) for 110 days with continuous ^14^C-CO_2_ labeling as described previously (Ge et al., 2012; Yuan et al., 2012a; Wu et al., 2014). The ^14^C-CO_2_ was generated by forcing a Na^14^_2_CO_3_ solution (1.0M, a radioactivity of 1.68 × 10^4^ Bqμg^−1^ C) into an acid bath (HCl, 2M) and the gas concentration (^14^C-CO_2_) was maintained at approximately 350 μL L^−1^. During the incubation period, all soils were illuminated by a parabolic aluminum reflector lamp with an intensity of about 500 mmol photons m^−2^ s^−1^ for 12 h each day (8:00 a.m.–8:00 p.m.). The day/night air temperature inside the chamber was maintained at 31 ± 1°C/24 ± 1°C and the relative humidity was kept at 80–90%. Soils from rice-rice plots remained flooded with a 1–2 cm water layer while those from rapeseed-corn plots were drained. Rice-rapeseed soils were also incubated under the waterlogged condition, due to rice being the crop plant for the following growing season in this cropping system. At the end of the 110 day incubation, the flooded water was removed and soils from 0 to 1, 1 to 5, and 5 to 17 cm depth intervals were sampled. The sectioned soil layers, each with four replicates, were divided into two sub-samples. One sub-sample was stored at 4°C for biochemical analysis while the other was kept at −70°C for molecular analysis. For each sectioned soil sample, the soil moisture content was measured immediately after sampling.

### Determination of soil properties

Soil pH was determined using a pH meter (Delta 320, Mettler-Toledo Instruments Ltd., China) at a 1:2.5 (w:v) soil-to-H_2_O ratio. Soil organic carbon (SOC) and total nitrogen (TN) contents were measured by dry combustion with a macro elemental analyzer (Vario MAX C/N, Elementar Analyse Systeme, Germany). Total phosphorus (TP) was determined using the Mo-Sb colorimeteric method (Lu, 2000). Clay content was measured using the pipette method and cation exchange capacity (CEC) was determined by titration (Rhoades, 1982; Müller and Höper, 2004). ^14^C-SOC (mg kg^−1^) was determined according to Wu and O'Donnell (1997), and^14^C-MBC (mg kg^−1^) was analyzed using the fumigation-extraction method (Wu et al., 1990). The amounts of ^14^C-SOC and ^14^C-MBC were calculated using the following formulas:

$$\begin{array}{l} {}^{14}C-SOC=F_{1}R_{s}/R_{p}W\\ 14C-MBC=F_{2}(R_{f}-R_{uf})/R_{p}W\;kc \end{array}$$

where F_1_ and F_2_ represent the factors to convert the counting volume (1 ml from 40 ml plus soil water volume in ml for F_1_ and 1 ml from 80 ml plus soil water volume in ml for F_2_); R_s_ and R_p_, radioactivity (Bq ml^−1^; blank counts omitted) for the trap solution and that for Na^14^_2_CO_3_ (Bq mg^−1^ C l^−1^) used to produce ^14^C-CO_2_ in the growth chamber; R_f_ and R_uf_, radioactivity (B_q_ l^−1^; blank counts subtracted for the extractants of the fumigated soil and unfumigated soil, respectively); W, the weight (kg) of digested soil on an oven-dry basis; Kc, the factor (0.45) converting measured ^14^C into biomass ^14^C (Wu et al., 1990).

### DNA extraction, clone library construction, and phylogenetic analysis

DNA was extracted in triplicate from 500 mg (fresh weight) soil from each independent replicate, using the FastDNA Spin Kit (BIO101, Qbiogene Inc., Carlsbad, CA) according to the manufacturer's protocol. The integrity and quantity of the extracted DNA were evaluated by standard agarose gel electrophoresis and a spectrophotometer (Nanodrop ND-1000, PeqLab, Germany). The *cbbL* gene fragments from one randomly chosen replicate of rice-rice and rapeseed-corn cropping systems were amplified using the same thermal profile as previously described by Wu et al. (2014). PCR reactions were set up as follows: 12.5 μl 2 × PCR MasterMix (Tiangen, China), approximately 50 ng soil DNA, and 0.1 μM of each *cbbL* primer, modified by Tolli and King (2005) per reaction. In order to show the reproducibility of our approach, the *cbbL* gene fragments from two replicate samples originating from the rice-rapeseed rotation were generated separately. Subsequently, PCR products were purified with an agarose gel DNA purification kit (Tiangen, China) and ligated into the pGEM-T Easy Vector System (Promega, Mannheim, Germany), and then transformed into *E. coli* DH5α-competent cells. Positive clones were sequenced at the Beijing Genome Institute (Beijing, China).

Clone sequences were grouped into operational taxonomic units (OTUs) based on 95% nucleotide sequence similarity using Mothur (Schloss et al., 2009). The OTUs primarily responsible for the differences in *cbbL*-carrying bacterial community among samples were identified based on the similarity percentage analysis (SIMPER) using PAST (Hammer et al., 2001). The representative nucleotide sequences of these OTUs were subsequently translated into amino acid sequences and aligned with closely related known sequences in GenBank using Clustal W (http://www.ebi.ac.uk/clustalw). If necessary, alignments were manually refined. The resulting alignment was used to construct a neighbor-joining tree using MEGA 5.0 (Tamura et al., 2011). Bootstrap analysis of 1000 replicates was conducted to estimate the robustness of the tree topologies.

### Community diversity analysis

Rarefaction curves were generated by the Analytic Rarefaction program (http://strata.uga.edu/software/Software.html) to assess the sampling effort. Shannon indices were computed using Mothur to compare the diversity of the *cbbL*-bearing bacterial communities in three cropping systems (Schloss and Handelsman, 2008). The coverage rate was computed as C = [1− (n/N)] × 100, where n represents the number of OTUs containing one individual sequence and N is the total number of sequences.

### Real-time PCR

The *cbbL* gene abundance was quantified using an ABI 7900 real-time PCR system (ABI 7900, Foster City, CA, USA) using SYBR Green I based assays. Quantitative PCR was performed in 10 μL reaction mixtures containing 5 μL 1 × SYBR Premix Extaq (Takara Bio Inc., Shiga, Japan), 5 ng of template DNA, 0.1 μM of primers with the following thermal profile: 30 s at 95°C, followed by 5 cycles of 5 s at 95°C, 45 s annealing temperature decreased from 66 to 62°C and an extension at 72°C for 30 s. In addition, another 35 cycles at 95°C for 5 s, 62°C for 45 s, and 72°C for 30 s. A final melting curve was generated to evaluate the amplification specificity. Ten-fold serial dilutions of plasmid DNA extracted from positive clones were used to establish a standard curve. The real-time PCR assays were performed in triplicate for each replicate sample. The copy number of the *cbbL* gene was calculated directly using SDS 2.3 software.

### RubisCO activity

RubisCO enzyme activity was assayed according to Yuan et al. (2012a). Briefly, 2 g soil (four replicates) were thoroughly homogenized by an ultrasonic cell mixer (JY92-II Scientz, China) in an extraction buffer containing Tris-HCl (100 mM, pH 7.8) and Dithioreitol (DTT, 1 mM). The supernatant was collected by centrifugation and was precipitated with solid ammonium sulfate to reach 80% saturation. The resulting pellets were collected and dissolved in Tris-HCl/DTT. RubisCO activity was measured at 30°C using spectrophotometry (UV-2450, Shimadzu, Japan) and calculated according to Takai et al. (2005).

### Statistical analysis

Canonical correspondence analysis (CCA) was performed using CANOCO 5.0 for Windows (Microcomputer Power, Ithaca, NY, USA) to characterize the effect of measured soil properties on the composition of bacteria communities carrying *cbbL* gene. Significant differences in community composition were tested by permutational Two-Way analysis of variance or multivariate analysis of variance (MANOVA) implemented in PAST (Hammer et al., 2001). PERMANOVA is a distance-based non-parametric MANOVA that allows the analysis of multivariate (or univariate) data in response to treatments in an experimental design. Statistical significant differences between data sets based on metadata (soil parameters, *cbbL* copy numbers, RubisCO activities) were identified by two-way analysis of variance (ANOVA) and differences were considered significant at *P* < 0.05. A multiple regression model was built by stepwise regression with significance being defined as *P* < 0.05. ANOVA and multiple regression analyses were carried out using SPSS (version 16.0, SPSS Inc., USA). The reproducibility of the carried out clone library analysis was tested using the aforementioned replicated clone libraries originating from the rice-rapeseed rotation. The robustness of clone library analysis was assessed based on calculated unweighted UniFrac distances (Lozupone and Knight, 2005).

### Nucleotide sequence accession numbers

Nucleotide sequences were deposited in the EMBL European Nucleotide Database (http://www.ebi.ac.uk/ena/data/view/) under accession numbers HG940678–HG941631.

## Results

### Incorporation of labeled ^14^C into soil organic matter and soil microbial biomass

The ^14^C-SOC and ^14^C-MBC concentrations were significantly different in the three cropping systems according to Two-Way ANOVA analysis. In 0–1 cm, the maximum ^14^C-SOC and ^14^C-MBC concentrations were detected in rice-rice rotated soil, reaching 935 mg kg^−1^ and 375 mg kg^−1^ respectively. The ^14^C-SOC and ^14^C-MBC concentrations in corresponding depths of rice-rapeseed rotated soil were 1.6 and 4.0 times lower than those in rice-rice rotated soil (Table 2). In 1–5 cm, the radioactivity, in terms of SOC and MBC was higher in rice-rice rotated soil than in rice-rapeseed rotated soil (Table 2). The amount of ^14^C incorporated in rapeseed-corn rotated soil was much lower compared to all other soils when investigating the 0–1 and 1–5 cm soil layers (Table 2). In addition, the ^14^C-SOC and ^14^C-MBC contents decreased with increasing depth, irrespective of the cropping systems. No radioactivity was detected in the 5–17 cm layer with the exception of rice-rapeseed rotated soil (Table 2). A statistically significant correlation was observed between ^14^C-SOC and ^14^C-MBC concentration (*P* < 0.05). The *cbbL* gene abundance and RubisCO activity were significantly related to ^14^C-SOC concentration (*P* < 0.05).

**Table 2:** **Amounts of ^14^C-SOC, ^14^C-MBC, abundance and activity of *cbbL*-carrying bacteria in three cropping systems**.

**Cropping system**	**Depth (cm)**	**^14^C-SOC concentration (mg kg^−1^ soil)**	**^14^C-MBC concentration (mg kg^−1^ soil)**	**Abundance (10^9^ copies g^−1^ dry soil)**	**Activity (nmol CO_2_ g^−1^ soil min^−1^)**
	0−1	934.97 ± 17.54 Aa	375.22 ± 5.19 Aa	2.59 ± 0.31 Aa	55.18 ± 7.82 Aa
Rice-rice	1−5	66.57 ± 3.05 Ab	27.88 ± 1.20 Ab	1.16 ± 0.19 Ab	48.84 ± 2.71 Aab
	5−17	ND	ND	0.66 ± 0.08 Ab	40.56 ± 1.02 Ab
	0−1	363.16 ± 117.64 Ba	73.03 ± 5.86 Ba	0.63 ± 0.11 Ba	43.64 ± 1.50 Ba
Rice-rapeseed	1−5	12.45 ± 1.12 Bb	4.37 ± 0.34 Bb	0.44 ± 0.06 Bab	30.69 ± 0.92 Bb
	5−17	3.71 ± 0.94 Bb	ND	0.25 ± 0.05 Bb	26.92 ± 0.68 Bc
	0−1	9.87 ± 0.41 Ba	3.91 ± 0.30 Ca	0.30 ± 0.13 Ba	29.13 ± 2.37 Ca
Rapeseed-corn	1−5	5.29 ± 0.44 Ba	1.70 ± 0.37 Ca	0.39 ± 0.14 Ba	27.55 ± 0.91 Cab
	5−17	ND	ND	0.36 ± 0.04 Ba	24.76 ± 0.55 Cb

*ND, not determined. Values are mean ± standard error in four replicates, different capital and low-case letters in the same column indicate significant differences (P < 0.05) among cropping systems and soil depths*.

### Diversity analysis of *cbbL*-carrying bacterial communities

**Figure 1:**
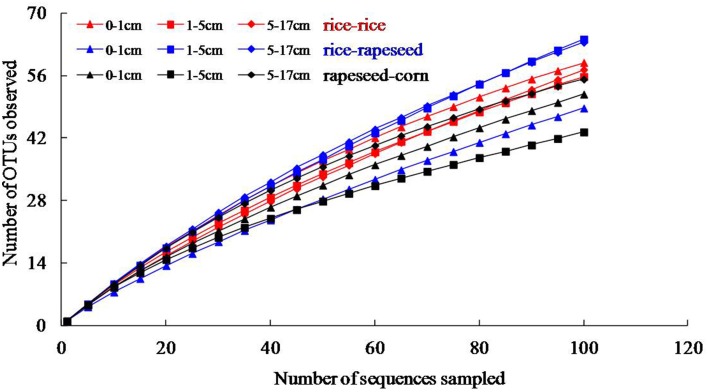
**Rarefaction analysis of *cbbL* clone libraries based on 95% nucleotide sequences similarity**.

Nine clone libraries of 106 clones each were obtained from different depth intervals for the three cropping systems. Overall, sequences were grouped into 148, 155, and 111 OTUs for rice-rice, rice-rapeseed and rapeseed-corn rotated soils, respectively (Table 3). High levels of diversity were observed based on the number of OTUs as well as calculated Shannon indices. Differences in diversity between cropping systems were small (Table 3). Rarefaction analyses suggested that additional sequencing effort is needed to cover the full diversity of *cbbL* sequences in our systems (Figure 1). The underestimated diversity was also reflected by the coverage of libraries, which ranged from 52 to 71% (Table 3). Determined Pearson coefficients revealed no significant correlations between diversity and monitored soil properties (*P* > 0.05).

**Table 3:** **Diversity of *cbbL*-containing bacterial community in sectioned soil depths from different cropping systems**.

**Cropping system**	**Depth (cm)**	**No. of clones**	**No. of OTUs**	**Shannon-Weiner (H)**	**Evenness**	**Coverage (%)**
	0−1	106	61	3.93	0.96	66
Rice-rice	1−5	106	59	3.76	0.93	63
	5−17	106	66	3.66	0.89	57
	0−1	106	51	3.18	0.81	63
Rice-rapeseed	1−5	106	67	3.95	0.94	52
	5−17	106	66	4.01	0.96	58
	0−1	106	55	3.56	0.89	64
Rapeseed-corn	1−5	106	49	3.40	0.89	73
	5−17	106	57	3.86	0.95	71

### *cbbL*-carrying bacterial community structures

**Figure 2:**
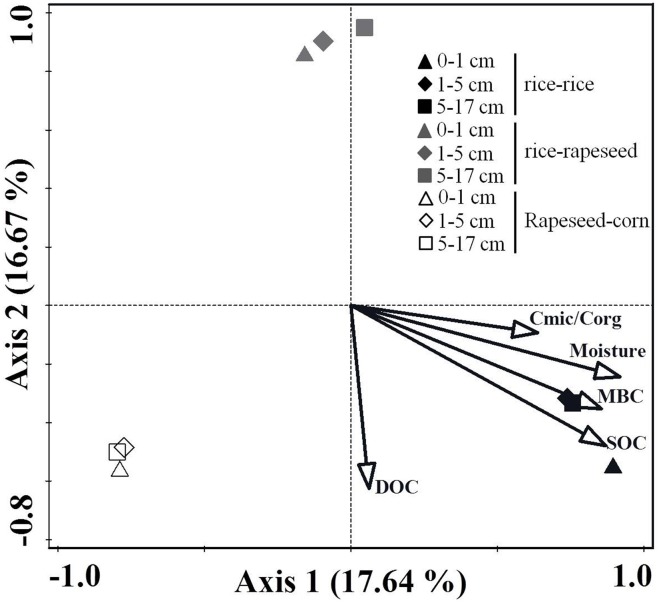
**Changes in the *cbbL*-containing bacterial communities in response to different cropping systems**. Cmic/Corg describes the ratio of microbial biomass carbon to total organic carbon.

The comparison of *cbbL*-bearing bacterial communities in two rice-rapeseed rotated soils revealed no statistically significant differences in community structures for replicated samples (Table S1), suggesting that our sampling approach is robust enough to draw reliable conclusions. The *cbbL*-carrying bacterial communities within the three cropping systems clustered into different groups, as revealed by the CCA analysis (Figure 2). Samples from different depth layers of the same cropping system formed relatively tight clusters (Figure 2). PERMANOVA analysis showed that the individual cropping system was a statistically significant determinant of community composition (*P* < 0.05), whereas the community structure did not change markedly at different sampling depths (*P* > 0.05). CCA analysis revealed that the SOC content (*P* < 0.05) was the main environmental driver for changes in the *cbbL*-bearing bacterial communities.

### *cbbL* gene abundance and RubisCO activity

The *cbbL* gene abundances in rice-rice rotated soil were significantly higher than those determined in rice-rapeseed and rapeseed-corn rotated soils (Table 2). Gene copy numbers were four times higher in the 0–1 cm layer, when compared to the 5–17 cm layer in rice-rice rotated soil (Table 2). Similar vertical trends were found for rice-rapeseed rotated soil, where copy numbers decreased by 63% in the 5–17 cm layer compared to the 0–1 cm layer (Table 2). *cbbL* copy numbers changed only slightly with depth in rapeseed-corn soil (Table 2). The abundance of *cbbL*-carrying bacteria was significantly correlated with DOC and MBC (*P* < 0.05).

RubisCO activity differed in the individual cropping systems. Highest activities were seen in rice-rice rotated soil (Table 2). Activities were generally found to decrease with soil depth. Multiple regression analysis based on stepwise showed that MBC was the main factor affecting RubisCO activity (*P* < 0.05).

### Phylogenetic affiliations of abundant OTUs

**Figure 3:**
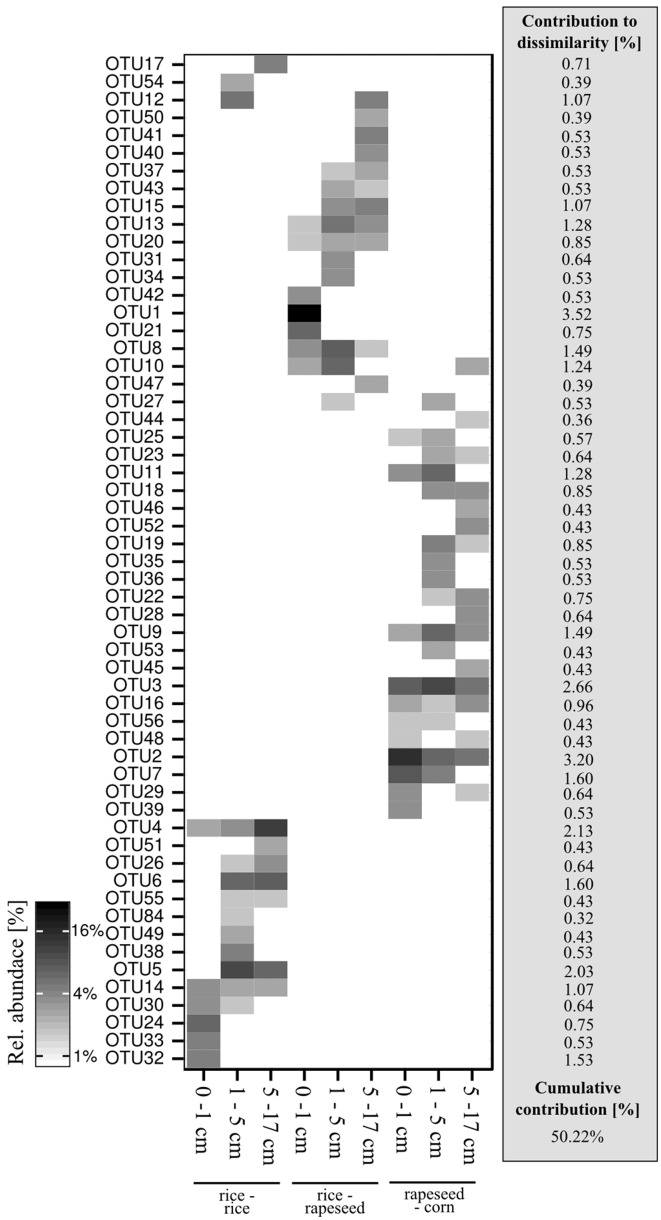
**Heatmap of the 57 OTUs primarily responsible for community differences**. Respective cumulative contributions to community dissimilarities are given in addition.

**Figure 4:**
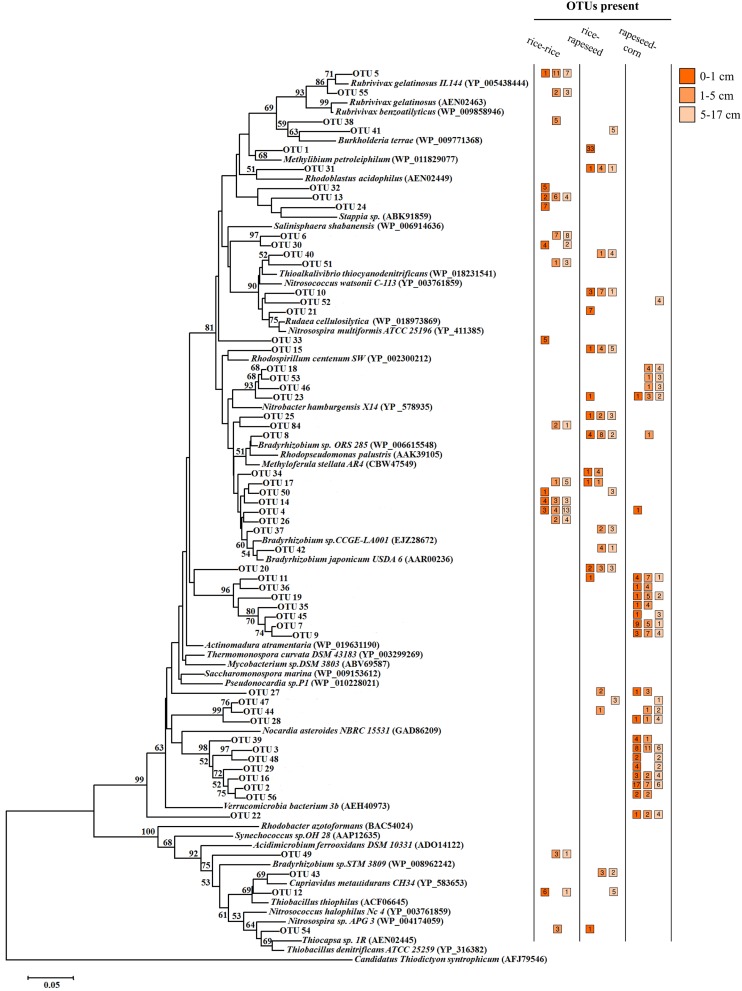
**Neighbor-joining tree illustrating the phylogeny of the OTUs primarily responsible for the differences in *cbbL*-carrying bacterial communities**. The tree was constructed using 165 deduced amino acids of corresponding nucleic acid *cbbL* clone sequences and 36 reference sequences from public databases. The *cbbM* gene from *Candidatus* “Thiodictyon syntrophicum” strain Cad 16 (accession number AFJ79546) was used as outgroup. The number of sequences retrieved from different samples is displayed in the colored squares next to the OTUs. Bootstrap values above 50% are indicated at the branch nodes. The scale bar represents 0.05 substitutions per amino acid based on a p-distance matrix analysis.

A total of 57 OTUs were identified as main phylotypes responsible for observed differences in community structure, cumulatively contributing 50% of the community variation (Figure 3). Sequences from these OTUs were dominated by facultative *cbbL*-carrying bacterial communities (Form IC), and they varied in their relative abundances in relation to cropping system and soil depth. Sequences from rapeseed-corn soil mainly formed four clades, with two clusters relating to *Nitrobacter hamburgensis* and *Nocardia asteroides* respectively and two novel clades without known representatives (Figure 4). Sequences from rice-rapeseed rotated soil were phylogenetically diverse, but closely related to sequences from *Methylibium petroleiphilum, Rhodoblastus acidophilus, Bradyrhizobium*, and *Cupriavidus metallidurans* (Figure 4). Sequences retrieved from rice-rice soil were widely distributed, grouping with various facultative and obligate autotrophic groups such as *Rubrivivax, Burkholderia, Bradyrhizobium, Stappia, and Thiobacillus thiophilus* (Figure 4).

## Discussion

According to our previous microcosm experiments, autotrophic bacteria contribute significantly to CO_2_ fixation in agricultural soils (Yuan et al., 2012a; Ge et al., 2013; Wu et al., 2014). We previously ascertained that the ^14^C incorporation, as a measure for the autotrophic soil carbon sink mediated by autotrophic bacteria, was 2–13-folds larger in continuous paddy rice soils than upland crop soils (Yuan et al., 2012a; Ge et al., 2013). Here we extended previous work to gain insights into the effect of different cropping systems on microbial CO_2_ fixation processes, using three different cropping systems, including rice-rice, rice-rapeseed and rapeseed-corn rotated soils. The significant linear correlation between ^14^C-SOC and ^14^C-MBC concentrations indicated that the fixed ^14^C in three cropping systems was derived from microbial fixation (Yuan et al., 2012a; Ge et al., 2013; Wu et al., 2014). Autotrophic bacteria, as revealed by the positive relationships between *cbbL* gene abundance, RubisCO activity and ^14^C-SOC concentration, were the major microbial players behind ^14^CO_2_ incorporation into SOC. The *cbbL*-carrying bacteria recovered in these soils were dominated by sequences related to facultative autotrophs like phototrophic, nitrogen fixing, nitrifying and CO and hydrogen oxidizing bacteria. However, some members of the *cbbL*-carrying bacterial communities were exclusive in one cropping system. For example, clone sequences in OTUs specific to rice-rice soil were closely related to *Rubrivivax gelatinosus*, which is a phototrophic bacterium with two *cbbL* gene copies surviving in aquatic ecosystems and moist soils (Kuske et al., 1997; Badger and Bek, 2008). While sequences closely affiliated to methylotrophic bacterium *Methylibium petroleiphilum* PM1 were exclusive to rice-rapeseed soil, whose presence has been documented in aquatic systems previously (Chen et al., 2009). Although facultative chemoautotrophy has been identified as an alternative metabolism in the methylotrophic bacterium *Beijerinckia mobilis*, a potential autotrophic metabolism of *Methylibium petroleiphilum* PM1 has not yet been demonstrated (Dedysh et al., 2005; Kane et al., 2007).

The diversity of *cbbL*-carrying bacterial communities suggested the presence of metabolically versatile autotrophic bacteria in the three cropping systems under study here. Much lower diversity patterns were reported in previous work regarding different managed agricultural soils using T-RFLP analysis (Selesi et al., 2005; Yuan et al., 2013; Xiao et al., 2014a). Applying clone library analyses improved the resolution of *cbbL* sequence analysis in comparison to previous studies (Marsh, 1999). Observed high diversities are presumably a consequence of changing underlying soil properties due to the applied cropping systems. Numerous studies have established a link between the *cbbL* diversity and soil properties (Nanba et al., 2004; Selesi et al., 2005; Yuan et al., 2012a,b, 2013; Xiao et al., 2014a). In our study, all the tested soils were developed from the quaternary red earth, which is characterized by a high clay content. The high amount of available nutrients in soil clay fractions were reported to favor the development of diverse *cbbL*-carrying bacterial communities (Paul et al., 2001; Selesi et al., 2007). This likely explains higher diversities in our soils in comparison to those observed in coastal barren saline soils based on clone library analysis (Yousuf et al., 2012).

The contributions of autotrophic bacteria to CO_2_ fixation in the three cropping systems were different, with the highest ^14^C incorporation rate observed in rice-rice, followed by rice-rapeseed, with the lowest value detected in rapeseed-corn rotated soil (Table 1). Paddy-upland rotation cropping systems differ from normal paddy rice and upland crop systems, where various water regimes are practiced in different crop growth seasons (Nishimura et al., 2008; Liu et al., 2010). During the submerged period of paddy rice cultivation, the anoxic conditions restrict the mineralization processes in soil, whereas the drainage of paddy fields for upland crop cultivation resulted in an oxic condition which enhances the decomposition processes within the soil (Chang Chien et al., 2006; Iqbal et al., 2009). As a result, physicochemical properties (e.g., SOC) are significantly altered in paddy-upland rotated systems when compared to paddy and upland soil traditional cultivation methods (Wang and Yang, 2003; Liu et al., 2010; Zhu et al., 2010). In this study, all soils were collected from the same climatic condition, and have the same origin and cultivation history. Soil properties such as SOC and TN were changing in response to the three cropping systems (Table 1), due to the differences in field management and crop regime over the 10 year period. The variations in soil properties (SOC, TN, MBC, and DOC) caused by the applied cropping systems affected the abundance, activity and composition of *cbbL*-carrying bacteria (Selesi et al., 2005; Xiao et al., 2014a), and thus resulted in the differences in ^14^C incorporation rates in soils. We observed an almost two orders of magnitude difference in ^14^C assimilation rates between rotation systems but only two- to four-fold differences in *cbbL* gene abundance and RubisCO activity. Considering that only *cbbL* copy numbers have been determined, differences on the level of transcription and translation cannot be ruled out. It appears reasonable to assume that ^14^C was at least partially assimilated by alternative CO_2_ fixation pathways, such as the reductive acetyl-CoA cycle that is known to be commonly active under anoxic conditions (Campbell and Cary, 2004; Nakagawa et al., 2005).

Labeling experiments as the one presented here are easily affected by the availability of unlabeled substrate. Dependent on the availability of unlabeled CO_2_, determined incorporation rates are eventually influenced due to a dilution effect. The availability of CO_2_ in soil pore space is strongly correlated with ongoing respiration processes, which are influenced by present organic substrates (Van Hees et al., 2005; Iqbal et al., 2009). Against this background our results appeared robust against the outlined dilution effect. The SOC content was highest in rice-rice soil, which presumably stimulated respiration. As a result CO_2_ availability would have been rather high, potentially diluting added ^14^C-CO_2_. Nevertheless, ^14^C incorporation was the highest in these soils.

In line with previous work, fixed ^14^C significantly decreased with soil depth in three cropping systems, indicating that a large proportion of microbially fixed ^14^C was restricted to the surface soil (Wu et al., 2014). Nishimura et al. (2008) reported that land use change from paddy rice to upland crop not only affected soil properties within the surface soil layer, but also caused changes within the deeper soil layers. Therefore, the differences in soil properties across soil depths may affect the availability of substrate (^14^CO_2_) and electron donors to autotrophic bacteria, resulting in changes in ^14^C fixation rates with soil depth (Jeffery et al., 2009; Kellermann et al., 2012; Wu et al., 2014). In addition, ^14^CO_2_ reduced to methane in the flooded surface soil could be accessed by methane oxidizing bacteria, which might in part explain the large assimilation in the surface of rice-rice and rice-rapeseed soils as well. It cannot be ruled out that incorporated ^14^CO_2_ was at least partially derived from CO_2_ fixation by soil algae, which eventually released metabolites that could have been processed by heterotrophic organisms. Nevertheless, soil algae were presumably playing a minor role. From previous work it is known that their abundance is at least one order of magnitude lower compared to autotrophic bacteria (Yuan et al., 2012a). Future isotope based work could help to gain an insights regarding the active community fraction consuming CO_2_ among *cbbL*-carrying bacteria and algae as a whole, since our community structure analyses revealed only minor changes for *cbbL*–carrying bacterial communities within different depths.

The present study showed variations in CO_2_ fixation by autotrophic bacteria in response to different cropping systems. Statistical analysis revealed higher CO_2_ assimilation rates in rice-rice than rice-rapeseed and rapeseed-corn rotated soils. Observed differences in soil parameters caused by the applied cropping systems lead to changes in *cbbL* abundance, activity and bacterial community structure, and thus resulted in differences in ^14^C incorporation rates in the three cropping systems. These results broaden our knowledge about the importance of autotrophic bacteria involved in the soil carbon sink. However, questions still remain, including the true extent of *cbbL* diversity. Here, next generation technologies such as high throughout sequencing appear represent promising follow up approaches, because the resolution of the analysis would substantially increase. Identifying active autotrophs involved in CO_2_ fixation by studying community gene expression would provide a better understanding about organisms playing major roles under different soil management conditions and how active organisms eventually interact with each other.

### Conflict of interest statement

The authors declare that the research was conducted in the absence of any commercial or financial relationships that could be construed as a potential conflict of interest.
