# Supplementary material for: Cropping systems modulate the rate and magnitude of soil microbial autotrophic CO2 fixation in soil
Source: Front Microbiol. 2015 May 8;6:379. doi: 10.3389/fmicb.2015.00379 (PMC4424977; doi:10.3389/fmicb.2015.00379)
Supplement: Supplementary file 1 [file Table1.DOCX]

**Supplementary Table**

Table S1 The comparison of *cbbL*-containing bacteria community structure in two replicates of rice-rapeseed soils

| Depth (cm) | Replicate* | UWScore | UWSig |
| --- | --- | --- | --- |
| 0–1 | A–B | 0.822 | > 0.001 |
| 1–5 | A–B | 0.758 | > 0.001 |
| 5–17 | A–B | 0.762 | > 0.001 |

*Replicate A and replicate B;

Significance defined as UWSi < 0.001
